# Supplementary material for: Content-rich biological network constructed by mining PubMed abstracts
Source: BMC Bioinformatics. 2004 Oct 8;5:147. doi: 10.1186/1471-2105-5-147 (PMC528731; doi:10.1186/1471-2105-5-147)
Supplement: Additional File 5 — The original Chilibot query results of the term "long-term potentiation (LTP)" and 22 other terms, limiting the latest references analyzed to the years 1990, 1995, 2000, and 2004. [file 1471-2105-5-147-S5.bz2 › chilibotAdditionalFile5/ltp2000/html/PI-3K_ATF.html]

 


 **PI-3K** and **ATF** 
  
Found 4 abstracts in PubMed,  **4 abstracts were retrieved and analyzed**.  


---

 Search Google  |
 PDF files only 
|  EDU domain only 

---

**Interactive relationship** (e.g. stimulation, inhibition, etc)

- Further, PDGF, PI 3 kinase  [ **PI-3K** ] , and Rac1 each activated the cyclin D 1 promoter at the cyclic adenosine monophosphate response element binding protein CREB activating transcription factor  **ATF**  2 binding site, as evidenced by expression of a CREB  **ATF**  2 reporter plasmid.  Ref: 11017907 Am J Respir Cell Mol Biol, 2000

- :-)

  **Parallel relationship** (e.g. studied together, co-existance, homology, etc.)

  - These results suggest that CD28 signaling leading to the c jun promoter involves acidic sphingomyelinase... to activate factors binding to the MEF2 and  **ATF**  sites.  Ref: 10403485 Mol Immunol, 1999
  - Finally, PI 3 kinase  [ **PI-3K** ]  and Rac1 induced CREB  **ATF**  2 transactivation were each inhibited by catalase.  Ref: 11017907 Am J Respir Cell Mol Biol, 2000
